# Health insurance benefit package in Iran: a qualitative policy process analysis

**DOI:** 10.1186/s12913-020-05592-w

**Published:** 2020-08-06

**Authors:** Efat Mohamadi, Amirhossein Takian, Alireza Olyaeemanesh, Arash Rashidian, Ali Hassanzadeh, Moaven Razavi, Sadegh Ghazanfari

**Affiliations:** 1 Health Equity ResearchCenter (HERC), Tehran University of Medical Sciences, Tehran, Iran; 2 Department of Health Management and Economics, School of Public Health, Tehran University of Medical Sciences, Tehran, Iran; 3 Department of Global Health and Public Policy, School of Public Health, Tehran University of Medical Sciences, Tehran, Iran; 4 National Institute of Health Research, Tehran University of Medical Sciences, No. 70, Bozorgmehr Ava., Vesal St., Keshavars Blvd, Tehran, 1416833481 Iran; 5 Information, Evidence and Research Department, Eastern Mediterranean Regional Office, World Health Organization, Cairo, Egypt; 6 Health Insurance Organization of Iran, Tehran, Iran; 7 Brandeis University, Waltham, MA USA

**Keywords:** Benefit package, Policy process analysis, Health insurance, Iran

## Abstract

**Background:**

Insufficient transparency in prioritization of health services, multiple health insurance organizations with various and not-aligned policies, plus limited resources to provide comprehensive health coverage are among the challenges to design appropriate Health Insurance Benefit Package (HIBP) in Iran. This study aims to analyze Policy Process of Health Insurance Benefit Package in Iran.

**Method:**

Data were collected through semi-structured interviews with 25 experts, plus document analysis and observation, from February 2014 until October 2016. Using both deductive and inductive approaches, two independent researchers conducted data content analysis. We used MAXQDA.11 software for data management.

**Results:**

We identified 10 main themes, plus 81 sub-themes related to development and implementation of HIBP. These included: lack of transparent criteria for inclusion of services within HIBP, inadequate use of scientific evidence to determine the HIBP, lack of evaluation systems, and weak decision-making process. We propose 11 solutions and 25 policy options to improve the situation.

**Conclusion:**

The design and implementation of HIBP did not follow an evidence-based and logical algorithm in Iran. Rather, political and financial influences at the macro level determined the decisions. This is rooted in social, cultural, and economic norms in the country, whereby political and economic factors had the greatest impact on the implementation of HIBP. To define a cost-effective HIBP in Iran, it is pivotal to develop transparent and evidence-based guidelines about the processes and the stewardship of HIBP, which are in line with upstream policies and societal characteristics. In addition, the possible conflict of interests and its harms should be minimized in advance.

## Background

Health Insurance Benefit Package (HIBP) are the health-care services covered by the government. Health systems use various priority setting mechanisms to define their HIBP [1]. For instance, the National Health Services –NHS- in the United Kingdom covers almost all services provided by public healthcare centres that are affiliated with the Department of Health [2, 3]. Whereas, the National Health Insurance- NHI- system in Germany develops the HIBP and restricts compensations to defined services that are included in the HIBP(s) [4]. Based on its health system, each country has its own mechanism of priority setting for policy coverage, through which a list(s) of services that are covered by the health insurance, so-called HIBP(s), is developed [5, 6].

By definition, developing a HIBP involves prioritization of healthcare services based on pre-defined indicators, during which, economic, clinical, and socio- political factors are considered [7]. Cost-effective and efficient development of a HIBP may face many challenges, particularly in the context of low and middle-income countries (LMICs). Similar to other settings, Iran’s health system has been facing a series of challenges in developing and implementing appropriate HIBP, i.e., lack of shared perspectives among policy-makers, insufficient transparent prioritizing criteria, ambiguous and unclear organizational structures and unsustainable resources [8, 9]. The Iranian Supreme Council of Health Insurance (ISCHI) is in charge of the process of decision-making for inclusion of a specific healthcare service into the basic insurance package. Conventionally, such decisions have been taken based on the bargaining power of various parties attending the ISCHI’s meetings. For instance, the insurance corporations mainly take into account the financial burden of services [10].

Developing a HIBP is politically hierarchical and largely contextual, which is associated with the health system structure, available budget and technical capacity of the stakeholders [4]. Hence, no universal method exists to fit all health systems. This study aims to investigate the policy processes of developing and implementing the HIBP in Iran. We will propose evidence-informed policy options to increase the efficiency and cost-effectiveness of the current HIBP. Using policy process (as one of the four dimensions of policy: content, process, stakeholders, and content) analysis, this article attempts to answer the following questions: how to identify problems that are related to the development and implementation of the HIBP; who is engaged in the policy development process; how to develop a HIBP-related policy; how to formalize policies that are related to the HIBP; how to implement these policies (HIBP development, making decisions process of HIBP); and finally, how to evaluate the HIBP in use.

### Setting

Iran’s health system is among very few that have merged medical education into service delivery. The Ministry of Health and Medical Education (MOHME) holds the stewardship of health system in Iran [11]. Enjoying an extensive network of over 60 universities of medical sciences (UMSs) across 31 provinces, the MOHME adminsters planning, service delivery, education, medicines’ supply and research in Iran.society. Health system finacing is mixed and mainly provided through public expenditure (51%). Social health insurance organizations pay for outpatient, inpatient and diagnostic services to about 90% of Iran’s population. Although the major payment mechanism is Fee for Service (FFS), capitation is also used at the Primary Health Care (PHC) level, where 99 services, 436 medicines and 48 laboratory services are provided. Besides, at the second and third levels of healthcare provision, mainly specialized hospitals, 3685 services, 2210 medicines, 404 consumables, 796 laboratory services, and 709 medical imaging services are covered. The ISCHI, affiliated to the MOHME, is responsible for strategic purchasing of health services.

## Methods

**Fig. 1:**
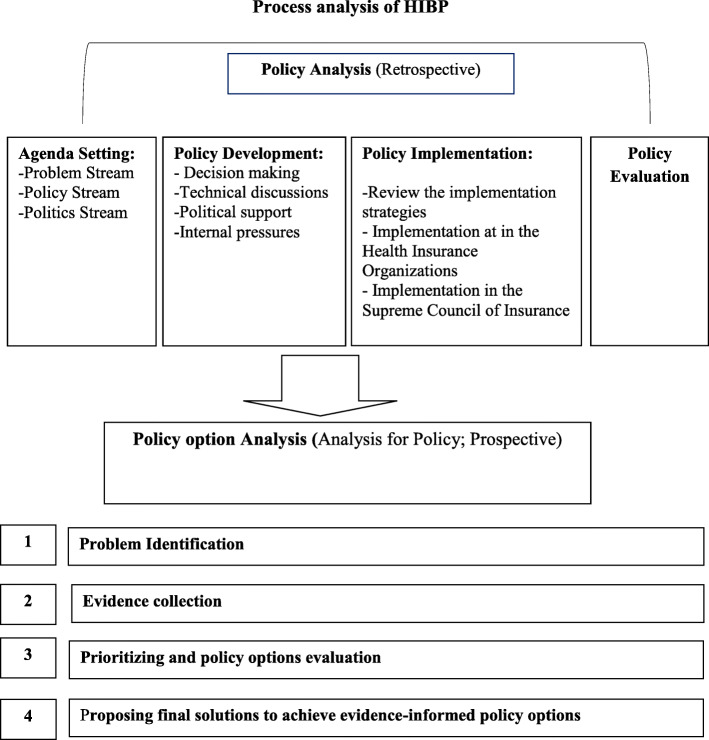
Conceptual framework of policy process analysis of HIBP in Iran

This is a qualitative research. We used both retrospective (policy analysis) and prospective (analysis for policy) approaches to investigate the policy-making process of the HIBP in Iran. “Policy analysis” refers to investigatation and analysis of past and current policies. “Analysis for policy” intends to identify appropriate policy options to address a challenge and improve policy [12]. Data collection and anlysis were conducted in two consecutive phases from February 2014 until October 2016. Conceptual freamwork of stydy is provided in Fig. 1.

### Phase 1: retrospective policy process analysis of HIBP

We investigated four dimensions of the policy process: agenda-setting, policy development, policy implementation, and evaluation. Our main method for data collection was face-to-face semi-structured interviews with purposefully identified experts (Appendix 1). The participants were senior managers of the MOHME, the Ministry of Cooperatives, Labor, and Social Welfare (MOCLSW), members of health insurance organizations and the ISCHI as well as informant academics in health financing, health insurance and health economics. Interviews were continued until we reached data saturation, when 25 expert were interviewed. In fact, in the last interviews, no new data was added to the study, so we concluded that the data was saturated. No one refused to participate or dropped out from interviews and we did not repeat any interviews.

We used a literature-based and tailored interview guide (Appendix 2). All interviews took place in the interviewees’ workplaces. The following issues were investigated during the interviews: how development of a HIBP was included in the MOHME agenda? How HIBP -related policies were developed (or are being developed)? The extent to which the HIBP development was evidence-based? What mechanisms were used to attract policy-makers’ attention to the HIBP -related problems? How HIBP -related policies are being implemented? Is there an evaluation and revision process for the HIBP? What instruments and solutions were used for revising the HIBP?

We also used documents review to collect data, including laws, instructions, and contents of various protocols that were related to the HIBP. We also developed an information worksheet to collect and categorize legal documents (Appendix 3) and to prepare them for thematic analysis.

In addition, one of authors (EM) participated in five meetings of the ISCHI, 15 h in total, to directly observe the decision-making process, stakeholders’ engagement and their influences. All discussions and the researcher’s perceptions were recorded.

We recorded all interviews and observations and transcribed verbatim. To ensure the accuracy of statements, we sent some transcripts to the interviewees and asked them for clarification, if necessary. Besides, relevant documents were categorized using the Microsoft Word software. An inductive thematic content analysis approach was used to analyze the data (Eloo 2007) and to categorize themes, MAXQDA.11 software was used to assist data management. AO and EM analyzed the data separately to assure the validity of the qualitative analysis.

### Phase 2: prospective policy-options analysis

We followed a four steps policy analysis model [13] to draw evidence-informed policy options about the issues and challenges of developing the HIBP:

1. **Problem identification:** The finding of phase one were used to identify and list the issues and challenges of each dimension.
2. **Evidence collection:** We collected scientific evidence for each identified issue through the following methods: comprehensive review of valid databases; experts’ opinions that were extracted from interviews; rationales extracted from investigating process; document review, and participating in ISCHI meetings. To search databases, MESH and Freetexts approaches were used. For this purpose, the most important medical electronic databases including the Cochrane, Pubmed, and Scopus were searched (2000-March 2016).
3. **Prioritizing and evaluating policy options:** after collecting evidence and primary development of policy options, a panel of professionals was convened to prioritize the policy options. A checklist which contained policy options (in the rows) and criteria (in the columns) was developed to obtain experts’ opinions. All identified options were evaluated in terms of feasibility and necessity. The participants were asked to rate each option on a Likert scale ranged from 1 (the worst) to 10 (the best) (Appendix 4).
4. **Final proposed solutions to achieve evidence-informed and prioritized policy options**: Experts’ opinions were analyzed based on specified criteria. The data from the previous phase were analyzed using the Simple Additive Weighting (SAW) method. Therefore, the total score of each policy option was calculated by multiplying the comparable rating for each criterion by the weight assigned to the criteria and then summing these values for all criteria. Data were analyzed using the Microsoft Excel software. Finally, we developed a summary of final solutions in the form of policy options.

## Results

In this section, first, we present findings of the retrospective qualitative analysis of the HIBP policies, followed by the results of policy options analysis.

Four main issues (i.e. agenda setting, policy development, policy implementation, and evaluation), 10 themes, and 78 sub-themes were identified (Table 1).

1. **Agenda setting**: To identify issues related to the Problem stream, Politics stream and Policies stream, the Kingdon multistream model was used [14]. Besides, 12 extra sub-themes were identified.

- **Problem stream**

**Table 1:** Policy “process” Analysis of HIBP

Issues	Themes	Sub-themes	
Agenda setting	Problem stream	1. Increasing the number of services that can be provided 2. Soaring health expenditures 3. Unavailability of information about inequality within insured populations 4. Inadequacy of resources 5. 5. Parallel budgets (insurances, hygiene, special programs, etc.)	
	Policies stream	6. Managing services that can be provided 7. Deficiencies in legislation and decision-making process that are related to the HIBP 8. Lack of clear criteria for including services in the HIBP 9. Not using professional and related staffs (not only those who are experienced) in implementation and support of the HIBP	
	Politics stream	10. Prioritizing health, and therefore its related policies, in the twelfth government 11. Increasing health sector budget in the 11th government 12. 13. Notifying OHP and making decision about the HIBP	
Policy development	Stewardship of the policy making	13. Developing the article 29 of the constitution 14. Developing policy’s draft by the MoHME and MoCLSW 15. HCHI as the steward of developing and notifying the HIBP’s strategies 16. Confirming policies by the National Expediency Council 17. Enacting policies by the Parliament 18. Final approval and notifying OHP by the supreme leader’s office 19. The MoHME is the steward of developing the HIBP based on the OHP	
	Method and trend of decision-making	20. Endorsing the HIBP by the third NDP for the first time 21. Lack of a defined methodology to include/exclude services into/from the HIBP 22. Drafted policies are different from notified policies, up to 70% 23. The ISCHI makes decision about the strategic policies of the HIBP 24. Developing polices according to the available resources 25. A defined contribution approach in developing HIBP-related policies 26. Inadequate attention to people’s preference/demand 27. 28. Using a top-down approach in developing HIBP-related policies in OHP	
Policy implementation	Policy implementation timeline	Before 1993	28. Article 29 of the constitution, requires the government to cover all necessary services 29. Lack of a clear distinction between service provision in public and private sectors 30. Lack of defined criteria to cover services by health insurance organizations 31. 33. Considering the availability of services when deciding to provide a service
		Between 1993 to 2003	32. Developing the UHI Act in 1993 and notifying it in 1994 33. Establishing the HCHI within the MoHME 34. HCHI became responsible about the HIBP 35. Experts debating in joint meetings 36. Commitment to provide all services that can be provided 37. Determining the covered services by the health insurance organizations 38. Political top-down decisions, without expert debates 39. Stakeholders or head of the meeting have greater influence
		2004 to 2006	40. Transferring the ISCHI from the MoHME to the MoCLSW 41. Insurance-related stakeholders had more influence 42. Services/medicines were included based on the frequency and compensation patterns 43. Including Services/medicines based on the reviewing less expensive services and equipment 44. Top-down political decisions, without expert debates 45. Introducing complementary insurance to cover services that were not covered by the basic insurance
		2007 to 2014	46. Developing the first comprehensive package 47. Using the most frequent services criterion to develop the HIBP 48. It takes a long time to decide whether to include a service/medicine or not 49. HCHI decides based on the consensus criteria 50. Special packages or separate resources/stewards (e.g. special diseases) 51. In 2010, the MoHME and the MoCLSW started strategic purchasing 52. New mandatory criteria were introduced (i.e. safety studies, effectiveness, cost-effectiveness) to include new medicines to the national formulary 53. In 2012, new RVU Book was developed
		Since 2014	54. In 2014, the OHP were notified by the Supreme Leader’s office 55. In 2014, the MoHME was mandated to develop the new HIBP 56. The MoCLSW was selected as the steward of financing and implementing the HIBP 57. In 2014, health transformation plan was started 58. The new HIBP was defined in the form of the RVU Book 59. Services that are not included in the HIBP were clearly mentioned in the new RVU Book 60. Defining and providing services that were not previously covered in the HIBP, as a part of the HTP
	Process of HIBP implementation	61. Sending a request to the ISCHI 62. Expert review of the request 63. Deciding about the request 64. If it has low financial burden, notifying its inclusion to the HIBP 65. If it has high financial burden, the cabinet confirmation is required	
Evaluation	HIBP Revision	66. Lack of fundamental and purposive revision(s) 67. Before 2014, there was no significant change occurred in the HIBP 68. Due to changes in the treatment methods, some services/drugs are automatically excluded 69. Mandating the ISCHI to annually revise the HIBP 70. Temporary and non-methodological changes (three times, in 2007, 2012, and 2014) 71. Unorganized revision of the OTC drugs 72. In 2003, some performance-enhancing drugs were excluded	
	Revising the methods and decisions	73. Process and criteria for including/excluding services are not revised 74. No evaluation has been performed, and laws and regulations are not revised 75. In 2013, service prioritizing program was begun, without clear outcomes	
	Evaluating the aims of HIBP-related policies	76. The impact of HIBP-related policies on achieving universal health insurance coverage 77. The impact of HIBP-related policies on developing basic and complementary HIBPs 78. The impact of HIBP-related policies on unifying the HIBP among all health insurance organizations	

The epidemiological transition fueled the constant increasing of demand for healthcare services, which led into spiraling health expenditures, which in turn revealed the importance of developing a HIBP. During the past four decades, a series of policies are developed and implemented in Iran that indicate the necessity of developing a basic health insurance package (e.g. the NHI Act of 1995, Supreme’s leader mega policies for health, and instruction of strategic purchasing):

*“Resource scarcity has always been an important problem for HIBP and, therefore, insurance organization always try to avoid implementing the HIBP …” (R 12).*

- **Policy stream**

Until now, no practical policy or scientific method is developed to design the implementation path of macro policies related to the HIBP in Iran. Issues such as lack of scientific criteria or evidence to develop or revise the HIBP and ignoring the epidemiological transition led into exacerbation of this problem:

*“Currently, our problem is that we mistakenly consider the HIBP as strategic purchasing, but it must be mentioned which services are covered, based on what evidences and for whom, and why this package should be bought, what criteria should be used, I mean, why a service should be included in the HIBP” (R 26).*

- **Politics stream**

In addition to political supports to HIBP that were endorsed by the sequential National Development Plans (NDPs), the Supreme leader’s mega policies for health (2013) were a turning point in providing political support for the HIBP. The mega policies attracted more attention to the health sector and led to allcation of extra funds towards the health sector:

*“In the eleventh government, government attention to the health sector problems and challenges significantly increased and continues” (R 11).*

Our investigation showed that HIBP -related policies have always been developing, but the three streams of problem, policy, and politics never came together. Inadequate systematic revisions and approaches to the HIBP resulted in insufficient growth of policies stream, which in turn prevented the policy window to become fully open.

2. **Policy development:** two main themes (stewardship of policy making, and method and trend of decision-making) and 15 sub-themes were identified.

- **Stewardship of the policy-making**

We identified 65 documents containing various policies that were, directly or indirectly, related to the HIBP. The most obvious one was Article 29 of the constitution, which endorses social security as a right for all citizens:

*“Having social security, in terms of retirement, unemployment, elderly, inability to work, orphanage, financial needs, accidents, health-care services and medical care, is a universal right for all Iranians” (Article 29 of the constitution).*

The MOHME is in charge of drafting health sector policies, while the MOCLSW contributes to developing the draft policies related to the HIBP. The MOHME is also responsible to get the policy approval in liaison with four levels: The ISCHI, the cabinet, parliament, and supreme leader’s office.

- **Methods and trends of decision-making**

The 3rd National Development Plan (NDP) of Iran endorsed health insurance, health system financing and HIBP -related issues for the first time, which were repeated in the next NDPs. Nevertheless, no organized decision-making process was designed to implement such policies. Consensus-making by officials and policy-makers (traditional negotiation style) was used to define the HIBP, where bargaining power had (and still has) an important role in influencing the decisions. The lack of transparency resulted in weak stewardship for HIBP-related policies:

*“A serious problem occurs in the system … because of the bargaining power of some policy-makers, some services won’t be included in the HIBP, while some unnecessary services are included, and it’s a serious problem in IHS” (R 6).*

3. **Policy implementation**: two main themes (policy implementation timeline and the process of HIBP implementation**),** plus 38 subthemes were identified here.

- **Policy implementation timeline**

On the basis of the changes in the content of the benefit package, decision-making method, and the stewardship of decision-making, the implementation and revisions of HIBP-related policies can be categorized into five periods: before 1993, 1994 to 2003, 2004 to 2006, 2007 to 2014, and after 2014. Before 1993 and the enactment of the Universal Health Insurance Act (UHIA), health laws were mainly focused on service coverage, whilst there was no comprehensive document to define the services that each health insurance organization should cover.

In 1993, by the enactment of the UHIA and establishment of the ISCHI, coherence of health insurance policies increased. The ISCHI was initially affiliated to the MOHME, while most of its members came from various health insurance organizations, plus the Iranian Medical Association (IMA). The ISCHI was responsible to make decisions about inclusion and/or exclusion of medical services into the HIBP. No debate among experts took place to make such decisions.

In 2004, the ISCHI was transferred to the newly established MOCLSW. During this period, the decision criteria to include new services were frequency and utilization patterns, which were based on the insurance organizations’ reports. In 2007, the biggest change occurred in the HIBP governance, when the ISCHI began to uniform the HIBP among all health insurance organizations. All covered services were published in a book, called “basic package of 2007”. After the enactment of the fifth NDP in 2012, the MOCLSW started a new reform to evaluate the HIBP. Although those measures were based on a scientific methodology –called “new HIBP”-, the previous package was enacted in reality.

The Health Transformation Plan (HTP) that was implemented in 2014 also affected the HIBP through revising the medical tariffs as well as the new Relative Value Unit (RVU) Book. In this book, all services that are available in Iran’s health system, i.e. procedures, surgeries, imaging, and laboratories are listed; those services which did not cover by any insurance organizations, are marked with an asterisk (*).

*“…By 2013, the book of RVU was published. This book includes all new and old health services. It was considered as a HIBP revision, the book was intended to revise the tariff but In fact, there was some kind of review HIBP…” (R 19)*

- **The process of the HIBP implementation**

Since 1993, all decisions about including and/or excluding a service within the HIBP are made by the ISCHI, with the participation of related stakeholders. When a new service is proposed to be included in the HIBP, the ISCHI invites various stakeholders (i.e. permanent members of the HHIC, and representatives of the MOHME, health insurance organizations, and the IMA as well as other members from professional associations), to attend in a meeting and to discuss the agenda. The process and methods of holding these meetings have not changed significantly ever since, with consensus building among members as the dominant method for making decisions. The bargaining power of health insurance organizations is mainly focused on the financial burden of services, while professional associations may attempt to exaggerate the importance of proposed services. Except for a few cases, no specific criteria and/or method (e.g. cost-effectiveness studies, guidelines) is used to make such decisions. As a rule, several meetings (in some cases it may take several years) are held to make a decision. Services with a high financial burden should be confirmed by the cabinet:

*“In some cases, health insurance organizations propose a service, all propositions, either from the MoHME or MoCLSW, send to the HCHI for expert analysis. There is a waiting list. Representatives from the different organizations as well as MoHME and MoCLSW debate. If consensus is on its inclusion, the cabined must confirm the decision” (R 3).*

4. **Evaluation of HIBP-related policies**: evaluation refers to the investigation of whether the goals of the policies were achieved and whether an implementation gap exists. Three main themes were identified: revision of the HIBP, revising the methods and decisions, and evaluating the aims of HIBP -related policies. 13 sub-themes were also identified.

- **HIBP’s revision**

Since 1993, any revision in the HIBP has been mainly focused on creating a more coherent and evidence-based package. In some cases (e.g. in 2007, 2012, and 2014), revisions were temporary and without a defined methodology. The findings showed that no purposive and fundamental revision was conducted. We identified a series of reactional, vs proactive, changes in the content of HIBP. Rarely, in less than 10 cases, an emerging need led to inclusion or exclusion of some medicines, medical equipment, and services into/from the HIBP:

*“It’s more than 30-years that we have the HIBP, but there is not a defined method for including a new and better service. Whether it should replace the older service or not”(R 4).*

Exclusion of over-the-counter (OTC) drugs was one of the main recent changes. In 2012, an expert panel was established for exclusion of OTC drugs from the HIBP and allocating the released funds for medicines related to special diseases.

- **Revising the methods and decisions**

Processes that are related to the inclusion and/or exclusion of services/drugs into/from the HIBP are not evaluated and revised yet. Meanwhile, due to technological advances or the introduction of lower-cost interventions, revisions deem necessary, some committed HIBP are not covered:

*“We never tried to revise the covered services. As well, we never tried to evaluate the HIBP” (R 12).*

- **Evaluating the aims of HIBP-related policies**

Despite the legislator’s emphasis on the annual revision of necessary commitments by health insurance organizations, this is only available for medicines packages and its execution was not regular. In 2007, Article 3 of the comprehensive welfare and social security system Act resulted in a big improvement towards a more transparent decision making about the HIBP and increasing the awareness about insurance services. According to the RVU Book (2015), coverage of inpatient and Para-clinic services included in the HIBP was 88 and 89.9%, respectively. Moreover, the National Health Accounts (NHA) (2013) showed that financial burden of uncovered services, those excluded from the HIBP, was only 6%.

### Limitations and solutions

After analyzing the interviews, fourteen challenges and constrains regarding the HIBP policies were identified. A summary of identified issues and problems is described in Table 2; it is worth noting that there are no priorities in the identified limitations.

**Table 2:** Limitations and problems of the HIBP policy process

**Limitations and issues that can be investigated**	
• Lack of clear criteria to include services into the HIBP • Not considering the epidemiological transitions to increase the effectiveness of included services. • Scientific evidences were not adequately used • Health Technology Assessment (HTA) studies were not used • Bargaining power had an important role in the ISCHI decisions • The extensive HIBP list regardless of the priorities and costs • Policies on HIBP and the strategic purchasing were not implemented • Cultural, social and economic issues were not considered • Passive performance of health insurance organizations to include new proposed services within the HIBP • Lack of revision and evaluation systems • OTC drugs are included in the HIBP • Unproportioned percentage of the health expenditures are created by a small percentage of patients • Development and implementation of programs and policies are not permanent • Inadequate resources	

11 solutions and 25 policy options were extracted, at least two policy options per each solution. Consequently, based on the pros and cons of each one as well as appropriateness and feasibility criteria, they were prioritized by an expert panel (Table 3).

**Table 3:** Solutions and policy options derived from the policy process analysis for the HIBP

Solutions	Policy options/description	Pros	Cons	Average Necessity and feasibility (+_) standard deviation (1–10)
Differentiating between HIBP(s) from services that can be provided	Defining necessary services benefit package and financing it by government and defining the higher level package that its financing is elective	Creating elective options for patients/ people and financial savings for the government	Establishing limitations on access to higher level services	7.8 ± 1
	Defining “necessary primary services HIBP” and financing it by the MoHME and also a “HIBP for secondary and tertiary necessary services” and financing it by insurance organizations	Ensure easy and free access to primary services, more effective management of curative services with stewardship of health insurance organizations	Inadequate attention of insurance organizations to the importance of preventive and screening services	5 ± 2.55
	Developing a HIBP that can be provided in all levels and financing it by health insurance organizations	Matching the HIBP with society’s health needs	Probability of increasing the number of covered services without considering available resources of health insurance organizations has increased	5.3 ± 2.3
Using scientific evidences to make HIBP-related decisions	Collecting and reviewing demographic information	Prioritizing services and evidence-based decision-making, indeed the HIBP should be targeted	Lack of precise information systems to determine the burden and pattern of diseases, by age groups	7.6 ± 1.5
	Conducting HTA studies	Developing a cost effective HIBP based on the comprehensive needs	These studies are cost driven and adequate experts to conduct them are not available	6.9 ± 1.6
	Considering cultural problems and needs in developing the HIBP (i.e. religious beliefs and cultural behaviors)	Increasing the acceptability of services for targeted populations, increasing equity in health	Increasing the probability of health expenditure soaring for the health system	4.6 ± 1.7
	Considering intervention’s QALY and DALY (analyzing the epidemiologic profile, and determining interventions based on it)	Prioritizing services that have more influence on life expectancy and quality of life	Ethical and social criteria are neglected	6.7 ± 1
Estimating the financial burden of diseases	Direct, indirect and intangible costs	Creating a systemic view or considering costs carried out by patients and avoiding catastrophic expenditures	Ignoring the necessity of covering some services that based on economic terms should not be covered	6.6 ± 1.6
Employing multi-criteria decision-making methods to develop the HIBP	Considering criteria that are related to economic aspects of services (cost effectiveness, budget impact, reducing poverty, quality and quantity of evidences and equity in better access to health-care services	More economic mix of services and avoiding exorbitant costs; transparency of definitions and prioritizing economic criteria	Some decision have unethical economic consequences	7.6 ± 1.1
	Mixing cost and effectiveness and economic and socio-economic criteria in related decisions (using multi-criteria decisions)	Creating a comprehensive view or considering all criteria that affects the decisions; increasing cost-effectiveness of the HIBP	Collecting information is time-consuming, and such decisions are costly	7.9 ± 1
Controlling inclusion of drugs, services and equipment that their effectiveness is not proved	The MoHME’s intervention in licensing new drugs and technologies or developing and implementing laws and regulations to restrict and control them	Increasing the control over services that can be provided, and, therefore, preventing the inclusion of services that are not cost effectiveness	A prolonged period is required to update health services of the country	8 ± 1.1
Organizing services/ drugs list that are covered or not covered	Developing a waiting list to include/exclude services/drugs (due to technological changes, policy change, new diseases patterns)	More efficient management of decisions to include/exclude services/drugs and facilitating annual revisions	More health human resources as well as continuous monitoring are required	8 ± 0.7
Creating a decision-making framework based on mathematical models and defined criteria	Weighting predetermined criteria and determining how to mix them by mathematical models	Transparency of method and process of decision-making and determining weights of criteria to make decisions	Possibility of conflict with ethical values in decision’s outcomes	6.7 ± 1
Expanding the package of services that can be provided	Expanding the HIBP by providing extra resources	Increasing access to health-care services	Services utilization is out of control and is creating exorbitant costs	5.8 ± 1.3
	Expanding the HIBP along with developing guidelines and standards for services provision	Increasing cost-effectiveness of services, reducing induced demand	Access to services can potentially be decreased	7 ± 1.2
	Expanding the HIBP along with developing specialized packages for each level of the health system	Increasing cost -effectiveness of services, reducing induced demand	Access to services can potentially be decreased	7.7 ± 1.2
Policies should be based on study’s findings and expert’s opinions	Macro decisions be made at higher levels and following that performing expert studies to increase efficacy of implementation	Clear tasks of middle and lower levels, converging tasks at lower levels	Environmental problems and issues are not reflected in macro decisions	7 ± 1.2
	Proposing policies by expert level and following that developing and notifying policies at macro level	Developing evidence-based policies	Prolonging decision-making process	7.3 ± 1.2
	Determining macro-level decisions orientation and following that developing expert-based policies	Transparency of overall strategies and finally making evidence-based decisions	Possibility of different interpretations that may be different from macro policies	7.9 ± 1.3
Organizing ISCHI meeting on including/excluding a service/drug/ equipment	Developing specialized forms which contain key criteria such as cost-effectiveness	Increasing efficacy of decisions through systematic process and defined participation of stakeholders	Challenges may arise in exceptional cases	8.3 ± 1
Revision and evaluation of the HIBP, both services-and- drugs related	Categorizing services/ drugs in three different lists (i.e. must be under coverage, can be covered, and must not be covered). Then, conducting cost-effectiveness studies for those services that can be covered	Making the HIBP cost-effective by spending minimum time and cost	HTA studies are not performed for all services; categorization may be biased	7.9 ± 1.3
	Conducting HTA studies for all services/drugs that can be provided, then revising the HIBP	Having a HIBP with cost-effective services, as much as possible	HTA studies are highly time and cost consuming; social criteria may be neglected	6.1 ± 1.6
	Perform the first method for the services in the package and the requirement for the HTA to include the new services / drug into the package	The HIBP will be cost-effective; these studies will be institutionalized in deciding about including services/ drugs	HTA studies are not performed for all services; categorization may be biased	7.5 ± 1.1
	Conducting second method and mandating HTA studies	Having a HIBP with highest possible of cost-effective services/drugs; these studies will be institutionalized in deciding about including services/ drugs	HTA studies are highly time and cost consuming; social criteria may be neglected	6.6 ± 1.8
	Determining the minimum expected level of health with measurable indicators to identify the situation or measuring the gap between coverage level and defined standards	Developing the HIBP based on the country’s needs	Lack of scientific evidences and field studies; conducing required studies require extra resources	5.8 ± 1.7

## Discussion

We investigated the policy process (i.e. agenda-setting, development, implementation, and evaluation) analysis for the HIBP in Iran. We found that various stakeholders developed different policies with different contents that had a defined algorithm. Meanwhile, different forces influenced the policy-making process. Such a mechanism has resulted in an idiosyncratic way of policy-making and defining the HIBP in Iran. At the macro level, the amount and source of financing are the main criteria to make such decisions.

According to the results, the main obstacle for inclusion or exclusion of services is lack of evidence-informed decision making. So far, several reforms have been conducted to revise the HIBP in Iran, the most important one was the third phase of the HTP that contained the revision of “Relative Values of the Diagnosis and Treatment Services”. It covers numerous diagnostic and surgery services that previously were not covered by basic insurance organizations. In the “Package for Reducing the Deduction for Diagnostic and Curative Services”, the co-payments for inpatient services were reduced from 10% (and informally about 30%) to 6%. This was accompanied by obliging the hospitals to provide all necessary equipment and supplies for patients within the hospitals. Implementing these policies caused substantial decline in absolute out-of-pocket payments for inpatient services. Nonetheless, further reforms are needed to improve strategic purchasing in Iran.

Our identified solutions and policy options showed that experts considered managing the inclusion of drugs, services, and equipment, organizing services/drugs lists, using scientific evidence to make HIBP -related decisions, and organizing ISCHI meeting on inclusion/exclusion of various items more than any other solution to define the HIBP. It seems that structural modifications are needed more than other changes to improve the HIBP.

The experience of other countries show the macro policy criteria, i.e. qualified services and diseases to be covered, ways to cover various age groups and financing methods, by both insurance organization and the government, as the main considerations in designing the benefit package [15]. In France, for example, an independent organization has been established to regulate, facilitate and enhance the transparency of the HIBP and organize providers’ compensation. A new treatment will only be accepted if it is proved to have higher benefits (with the same level of costs) or lower costs (with the same level of benefits) [7, 16]. It seems that the debates around developing policies and changing the steward of developing the HIBP are mostly focused on the source of financing, while adequate attention has not been paid to how to develop the HIBP with targeting diseases/individuals.

Several studies have investigated the concept of HIBP, its challenges and limitations. Studies performed in Colombia and Philippine used the instrument developed by WHO to assess the strategic purchasing of health services. In Colombia, the revision of the benefit package was reported to be based on a transparent, scientific-technical and participatory process [17]. Similar to Iran, in the Philippine, there was no benefit expansion plan or strategy. Hence, all the existing benefit packages of the Philippine might be crafted and approached in an unstandardized and ad hoc environment [18].

Another study conducted in Iran reported that one of the main challenges in the SP is the type of services and goods purchased (what to purchase?). In addition, they identified several problems in the present benefit package, i.e. inappropriate information systems, unsuitable mechanisms and criteria to select included services, and inappropriate trustees to decide about the service package. Simple interventions (i.e. prioritizing the services, determining the effectiveness, efficiency and safety of services, and definition of the criteria for reviewing the package as well as assessing the feasibility of introducing some preventive services to the package) can make the HIBP more effective [19].

The decision-making process to design HIBP is based on reliable evidence and through scientific methods in many countries [20]. Our findings revealed that the HIBP is mostly defined based on negotiating with stakeholders in Iran, while the HIBP revisions were mostly temporal and non-systematic. Evidence shows the need for systematic annual or at least biannual evaluations for substituting less effective services/drugs with more effective ones. This can increase the quality of provided services as well as efficiency. Thailand uses a four-step mechanism to make decisions that are related to include a service into the HIBP. They use the criteria as follows: the number of patients who suffer from the disease, severity, cost-effectiveness of intervention(s), types of available services, the economic impact on households, and ethical and equity issues in evaluating the package [21]. Norway and France use below criteria to evaluate a service: cost-effectiveness, personal benefits and severity of the disease [22].

### Policy recommendation

Here, following prioritization and evaluation of political options, we recommend:

- **Creating different packaged based on the type of services**

A HIBP should only contain ‘necessary services’, while other services can be financed through complementary insurance or users’ direct contribution.

- **Evidence-based decisions for the content of HIBP**

To incorporate evidence-informed decision-making criteria, i.e. Health Technology Assessment (HTA) and cost-effectiveness analysis, into the process of the ISCHI meetings, HIBP-related decisions should be based on scientific evidence, precise demographic information (separated by age groups, special needs of each age group, and defined targeted package according to such information) as well as considering a combination of cost-effectiveness and socio-economic conditions of the country (using multi-criteria decision-making to include services) in the frame of using multi-criteria decisions.

To control provision of services and procedures, a series of interventions and regulations should be introduced to restrict the inclusion of new drugs and technologies to the most cost-effective ones.

- **Periodical Revision of the HIBP**

In line with periodic evaluation and to increase the organization of services/drugs lists that are covered, a waiting list needs to be developed for those services/drugs that are under review to be included and those that are about to be excluded,. To increase the capacity of the health system for expansion of service provision based on the health equity and promoting Universal Health Coverage (UHC), new guidelines and standards should be developed for revising the HIBP. For instance, the coverage should be restricted to those who are eligible. Moreover, specialized HIBPs for each level of service provision based on the age groups and disease categories should be defined.

For revision and evaluation of the current HIBP, we suggest categorizing services and drugs into three different lists (i.e. must be covered, must not be covered, and can be covered) based on the cost-effectiveness, budget impact, safety, and availability of alternative services criteria as well as experts’ and users’ opinions. This can be galvanized by including the findings of HTA studies for the services that can be covered.

### Strengths and limitations

To the best of our knowledge, this is the first deep and extensive study for analyzing the HIBP policies in Iran, whose findings can respond to long-waiting questions of health policy-makers in this regard. The final solutions presented in this study are based on scientific and objective evidence that have been approved by the experts. However, our study had some limitations. We did not find a universal definition of a HIBP, and encountered discrepancies between scientific literature and the experience of different countries. We also faced some challenges in obtaining some documentation from different organizations, i.e. the executive instructions and the expired regulations that were not cited on the websites, due to which determining the effects of the HIBP implementation in achieving desired goals might be incomplete.

## Conclusions

Given the limited resources and ever-increasing public demand for healthcare services, designing an evidence-based HIBP, which is in line with upstream policies, is crucial to reach and sustain UHC in Iran. This renders a systematic implementation process and appropriate ways to manage stakeholders’ power and influence for minimizing the possibility of conflicts during the HIBP development. Equitable and quality healthcare with no one left behind is at the heart of UHC, which is in turn the center of sustainable health development. To reach UHC by 2025, as manifested by the MOHME, Iran has no choice but to implement substantial reforms into its pathway in designing evidence-informed health HIBP, i.e. but not limited to employing efficient financial, economic and political solutions, e.g. HTA. Unless the conventional method of negotiation and bargaining is replaced with robust, transparent, and culturally accepted ways of defining the HIBP, the healthcare system of Iran will face unsustainability in the provision of resources and public dissatisfaction, which may in turn endanger its pathway along with sustainable health development.

## Supplementary information

**Additional file 1.**

## Data Availability

The data of this study were raw data, which we access them by interview and report them in the paper. The datasets used and/or analyzed during the current study are available from the corresponding author on reasonable request.

## Abbreviations

HIBP: Health Insurance benefit Package
HTA: Health Technology Assessment
HTP: Health Transformation Plan
IMA: Iranian Medical Association
ISCHI: The Iranian Supreme Council of Health Insurance
MOCLSW: Ministry of Cooperatives, Labor, and Social Welfare
MOHME: Ministry of Health and Medical Education
NDPs: National Development Plans
NHA: National Health Accounts
NHI: National Health Insurance
OTC: Over-The-Counter
PHC: Primary Health Care
RVU: Relative Value Unit
SAW: Simple Additive weighting
UHC: Universal Health Coverage
UHIA: Universal Health Insurance Act
UMSs: Universities of Medical Sciences
