# Supplementary material for: Health insurance benefit package in Iran: a qualitative policy process analysis
Source: BMC Health Serv Res. 2020 Aug 6;20:722. doi: 10.1186/s12913-020-05592-w (PMC7409638; doi:10.1186/s12913-020-05592-w)
Supplement: Supplementary file 1 — Additional file 1. [file 12913_2020_5592_MOESM1_ESM.docx]

**Appendix1: List of Participants**

| **Organization** | **Education** | **Work record** |
| --- | --- | --- |
| Ministry of Health and Medical Education | MD.PHD | 6 |
| Ministry of Health and Medical Education | PHD | 5 |
| Ministry of Health and Medical Education | MD | 15 |
| Ministry of Health and Medical Education | Master of science | 17 |
| Ministry of Health and Medical Education | Bachelor | 7 |
| Ministry of Health and Medical Education | PHD | 4 |
| Ministry of Health and Medical Education | MD.PHD | 20 |
| Ministry of Health and Medical Education | PHD | 3 |
| Health insurance organization | MD | 17 |
| Imam Khomeini Relief Committee (Insurance) | Bachelor | 13 |
| Armed Forces Health Insurance | MD.PHD | 21 |
| Social Security Organization | MD.PHD | 9 |
| Social Security Organization | MD.PHD | 11 |
| Social Security Organization | Pharmacist | 12 |
| Armed Forces Health Insurance | MD | 30 |
| Health insurance organization | MD.PHD | 20 |
| Health insurance organization | MD.PHD | 17 |
| Islamic Consultative Assembly | MD- Specialists | 7 |
| Presidential Administration and Planning Organization | MD.PHD | 5 |
| Ministry of Welfare / the Iranian Supreme Council of Health Insurance | Dentist | 10 |
| Medical Council | MD.PHD | 20 |
| Baqiyatallah University of Medical Sciences | PHD | 15 |
| Tehran University of medical Science | MD.PHD | 20 |
| Tehran University of medical Science | MD.PHD | 3 |
| Tehran University of medical Science | Pharmacist. PHD | 12 |
| **Total number** | | **25** |

**Appendix 2: Interview Guide**

- Introduction of the project to the interviewee
- Provide an explanation of the subject of the project and its history to the interviewee
- The first question will be that after the brief introduction of the interviewee, the level of education, the field of work and the current position, what is their opinion on Health Insurance Benefit Package (HIBP)policies
- What is the HIBP policies process in Iran? (Steps taken to formulate and implement this policy, and to state the process of changes).
- How were sat HIBP policies in agenda?
- How were developed HIBP policies?
- How did HIBP policies get onto the policy agenda?
- How was the policy formulated?
- What was the policy style of HIBP?
- How far was decision-making in the hands of policy elites? How many times were consultations made? When?
- Planning or problem solving? Consultation or imposed?
- What were the negotiation styles amongst the policy elites?
- How much evidence was used for decision-making? Was it consensual?
- Was there any consultation with providers? Was there any negotiation between providers? How was it undertaken?
- Was there any measurement of health problems regarding HIBP policies in the system?
- Is the information available in annual reports?
- Who conducted the measurement of indicators?
- How did the mechanisms serve to bring problems to the attention of policy makers?
- Was there any mechanism in the system to feedback the failure of the government
- Performance such of the Health Welfare Scheme?
- Do the public support this policy?
- • Do politicians support this policy? How? • Which factors made HIBP acceptable to the political party?
- Technical feasibility? • Legitimacy? • Reformer commitment? • Empirical research on problems and solutions? • Economic crisis? Public interest?
- Who is involved in the implementation process?
- Is there extensive participation?
- Is implementation by the Ministry of Health only?
- Is there a clearly stated policy goal by the Minister?
- What did the implementers at peripheral level perceive as the policy goals?
- Was HIBP clearly stated?
- Was the policy planned to be introduced in a short time?
- Did implementation take place quickly? Why?
- Was there any resistance?
- What are the obstacles and challenges in implementing of HIBP?
- What parts of the implementation policy are implemented and which ones are not implemented?
- Will the HIBP policy be evaluated and reviewed?
- What are the tools and strategies for revising the HIBP?
- What individuals and organizations play a role in reviewing and evaluating of HIBP policies?
- Do you have any suggestions for promoting this policy?

**Appendix 3: worksheet information of document**

| **Worksheet information of document** | |
| --- | --- |
|  | Title |
|  | Type |
|  | Date of delivery |
|  | Author |
|  | The main audience |
|  | Text |

**Appendix 4: The format of checklist to evaluate policy options of HIBP**

| **Solutions** | **Policy options/description** | **Pros** | **Cons** | **Necessity**  **(1-10)** | **Feasibility**  **(1-10)** |
| --- | --- | --- | --- | --- | --- |
|  |  |  |  |  |  |
